# AMP-activated protein kinase: a potential therapeutic target for triple-negative breast cancer

**DOI:** 10.1186/s13058-019-1107-2

**Published:** 2019-02-21

**Authors:** Wei Cao, Jieqing Li, Qiongyu Hao, Jaydutt V Vadgama, Yong Wu

**Affiliations:** 1 Department of Nuclear Medicine, Union Hospital, Tongji Medical College, Huazhong University of Science and Technology, Wuhan, 430022 China; 2 Division of Cancer Research and Training, Department of Internal Medicine, Charles R. Drew University of Medicine and Science, David Geffen UCLA School of Medicine, and UCLA Jonsson Comprehensive Cancer Center, 1748 E. 118th Street, Los Angeles, CA 90059 USA; 3 Department of Breast Surgery, Tianjin Central Hospital of Gynecology and Obstetrics, Tianjin, China

**Keywords:** Triple-negative breast cancer, AMP-activated protein kinase, Chemotherapy, Targeted treatment

## Abstract

Triple-negative breast cancer (TNBC) is an aggressive subset of breast carcinomas that lack expression of estrogen receptor (ER), progesterone receptor (PR), and human epidermal growth factor receptor-2 (HER2). Unlike other breast cancer subtypes, targeted therapy is presently unavailable for patients with TNBC. In spite of initial responses to chemotherapy, drug resistance tends to develop rapidly and the prognosis of metastatic TNBC is poor. Hence, there is an urgent need for novel-targeted treatment methods or development of safe and effective alternatives with recognized mechanism(s) of action. AMP-activated protein kinase (AMPK), an energy sensor, can regulate protein and lipid metabolism responding to alterations in energy supply. In the past 10 years, interest in AMPK has increased widely since it appeared as an attractive targeting molecule for cancer therapy. There has been a deep understanding of the possible role of abnormal AMPK signaling pathways in the regulation of growth and survival and the development of drug resistance in TNBC. The increasing popularity of using AMPK regulators for TNBC-targeted therapy is supported by a considerable development in ascertaining the molecular pathways implicated. This review highlights the available evidence for AMPK-targeted anti-TNBC activity of various agents or treatment strategies, with special attention placed on recent preclinical and clinical advances in the manipulation of AMPK in TNBC. The elaborative analysis of these AMPK-related signaling pathways will have a noteworthy impact on the development of AMPK regulators, resulting in efficacious treatments for this lethal disease.

## Background

Breast cancer (BC) is the most common malignant tumor in women [1]. Approximately 70% of BC patients express estrogen receptor-α (ERα). Because of the success of endocrine therapy, the mortality rate of patients with ERα+ cancers has decreased dramatically. Likewise, around 15% of patients have cancers overexpressing human epidermal growth factor receptor-2 (HER2) and hence are candidates for HER2-targeted treatments. Conversely, triple-negative breast cancer (TNBC) represents cancers lacking clinical expression of ERα, progesterone receptor (PR), and HER2 (ER-/PR-/HER2-) and cannot be treated with current endocrine or HER2-targeted therapies. This type of cancer overlaps partially with basal-like BC, a subgroup that expresses specific cytokeratins, and some hereditary BCs. Furthermore, this subtype is associated with undesirable biological characteristics, such as high mitotic count and aggressive behavior. While TNBCs only occur in 10–15% of patients, they account for almost half of all BC deaths. Despite the heterogeneous nature, TNBCs frequently occur in African American [2] and younger women [3] and among patients with *BRCA1/2* gene defects [4]**.**

Although the term “TNBC” has only recently appeared in the medical literature, it has acquired such a degree of scientific interest that the category of TNBC has now been fully integrated into the terminology of oncology. Nowadays, TNBC may be one of the most active fields in oncology research due to the following reasons: (1) In the context of the current treatment of BC, there is a lack of accepted molecular therapeutic targets, making TNBC a new orphan disease; (2) The prognosis of TNBC patients is comparatively poor, particularly in advanced patients, making TNBC a very challenging and disheartening situation for patients and medical oncologists [3]. In view of the malignancy of TNBC and the mortality rate of those with metastatic BC, further studies are needed to improve the prognosis of this subtype of BC.

### TNBC treatment

TNBC treatments consist of two parts, namely locoregional treatments, including surgery and radiotherapy, and systemic treatments, such as chemotherapy and targeted therapy. Compared with locoregional treatments, systemic treatments are directed toward genetic aberrations and the molecular status of tumors. The preferred cytotoxic chemotherapy regimens for primary TNBC are mainly based on taxane, anthracycline, and sometimes cyclophosphamide, while several combination therapies including methotrexate and epirubicin could be considered [5]. In general, TNBC is more sensitive to chemotherapy than any other subtype [6], and pathological complete response (pCR) can be achieved in 20–30% TNBC cases that received neoadjuvant chemotherapy [7]. Although improvements of pCR observed in TNBC result in prolonged overall survival (OS)/disease-free survival (DFS) [8], TNBC is still prone to metastasis and recurrence due to its heterogeneity [9]. For recurrent and metastatic BC, preferred chemotherapy agents include doxorubicin, paclitaxel, anti-metabolites (capecitabine and gemcitabine), and microtubule inhibitors (vinorelbine and eribulin), while cyclophosphamide, carboplatin, docetaxel, cisplatin, epirubicin, ixabepilone, and combination therapy could be treated as additional options [5].

Targeted therapy seems to be a potential solution for TNBC, and a number of antagonists, inhibitors, activators, and monoclonal antibodies have been put into preclinical and clinical trials (reviewed in [10]). The targets of these new drugs include androgen receptor (AR), poly (ADP-ribose) polymerase (PARP), cyclin-dependent kinases (CDKs), checkpoint kinase 1 (CHK1), DNA (cytosine-5)-methyltransferase 1 (DNMT1), epidermal growth factor (EGF), EGF receptor (EGFR), fibroblast growth factor receptor (FGFR), vascular endothelial growth factor (VEGF), VEGF receptor (VEGFR), p53, PI3K/AKT/mammalian target of rapamycin (mTOR), SRC, Wee1, and WNT. Up until now, most of these treatment options have not achieved satisfactory therapeutic results and olaparib, a PARP inhibitor, is the only one that has been recommended to treat BRCA1/2-positive recurrent or metastatic TNBC [5].

### AMPK in human TNBC

AMP-activated protein kinase (AMPK), a crucial metabolic sensor that can regulate energy homeostasis at the cellular and whole body levels, is an important hub between metabolism and signaling networks. Fifteen years ago, the tumor suppressor liver kinase B1 (LKB1) was found to be the main upstream kinase of AMPK, implying that the tumor suppressor effects of LKB1 may be mediated by AMPK [11]. Since then, AMPK-regulating drugs have been studied in vitro and in vivo to analyze the role of AMPK in carcinogenesis and progression of cancer. Studies examining the potential relationship between AMPK and its clinicopathologic significance in BC reveal that the expression levels of AMPK are relatively higher in TNBC versus non-triple-negative breast cancer (NTNBC) cell lines and that AMPK is also upregulated in TNBC tissues compared to NTNBC tissues [12]. Expression of AMPK is correlated with TNM stage, distant metastasis, and Ki67 status. Patients with positive expression of AMPK exhibit shorter OS and DFS [12]. These findings implicate AMPK as a promising prognostic biomarker for TNBC. Recent evidence has demonstrated that pAMPK is reduced by approximately 90% in cancer tissues of two cohorts of 354 primary BC patients versus normal breast epithelial cells [13]. Moreover, in both cohorts, decreased AMPK phosphorylation is strikingly related to higher histological grade and axillary node metastasis. Taken together, these results demonstrate that AMPK function is compromised in primary BCs. Decreased AMPK signaling and the negative correlation with histological grade/axillary node metastasis implicate that AMPK reactivation has the potential for prevention and treatment in BC.

**Fig. 1:**
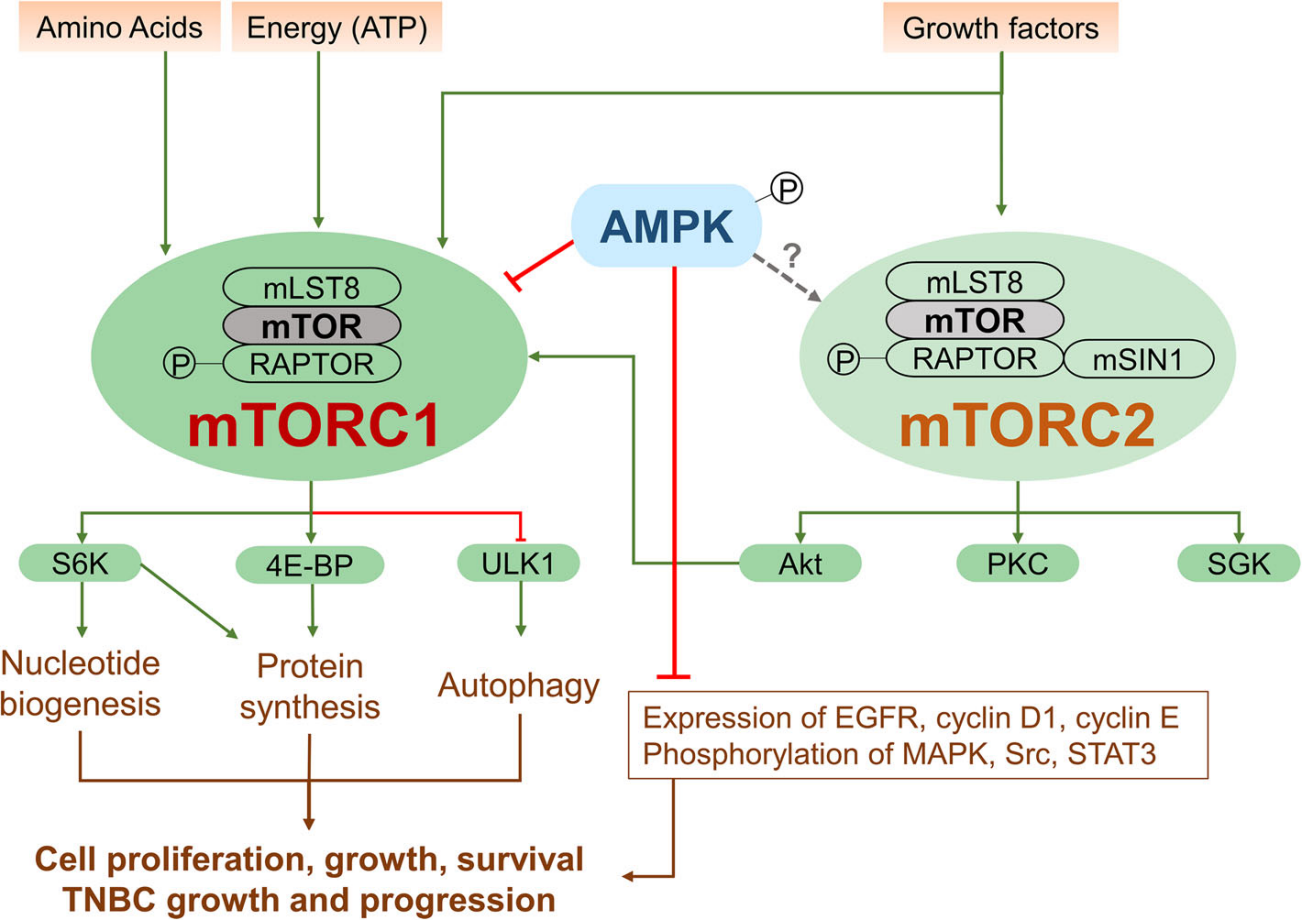
Schematic illustration of the AMPK/mTOR signaling pathway in TNBC tumor growth and progression. mTORC1 comprises mTOR, mammalian lethal with sec-13 protein 8 (mLST8), and regulatory-associated protein of mammalian target of rapamycin (RAPTOR). mTORC1 is activated by growth factors, nutrients (amino acids), and cellular energy. It stimulates anabolic processes, including protein and nucleotide synthesis via ribosomal protein S6 kinase (S6K), eukaryotic translation initiation factor 4E (eIF4E)-binding protein 1 (4E-BP1), and hinders catabolic processes, such as autophagy, through Unc-51-like kinase 1 (ULK1). mTORC2 consists of mTOR, mLST8, mammalian stress-activated map kinase-interacting protein 1 (mSIN1), and rapamycin-insensitive companion of mTOR (RICTOR) and is activated by growth factors. mTORC2 activates the AGC kinase family members Akt, serum/glucocorticoid-regulated kinase (SGK), and protein kinase C (PKC). mTORC1 and mTORC2 are commonly activated in human cancers. AMPK activation inhibits mTORC1; however, the effect on mTORC2/Akt is not completely clear. Moreover, AMPK activation represses expression of EGFR, cyclin D1, and cyclin E and phosphorylation of MAPK, Src, and STAT3

Given that the molecular pathology of TNBC including the pathogenesis of the disease and the resistance mechanisms to existing therapies is not known, it is problematic to develop new drugs that effectively target TNBC. In the past few years, there has been a deep understanding of the possible role of abnormal AMPK signaling pathways in the regulation of growth and survival and the development of drug resistance in TNBC. AMPK activation has positive effects in TNBCs due to its effect of target inhibition on Akt/mTOR [14]. Furthermore, AMPK activation represses expression of EGFR, cyclin D1, and cyclin E and phosphorylation of mitogen-activated protein kinase (MAPK), Src, and signal transducer and activator of transcription 3 (STAT3) [15, 16]. These results provide additional information that is likely to influence the development of TNBC targeted therapy (Fig. 1). Here, we summarize the available evidence for AMPK-targeted anti-TNBC activity of various agents or treatment strategies and attempt to give a prospect on targeted therapeutic strategies in the future.

### Metformin

Metformin is a first-line treatment for type 2 diabetes mellitus (T2DM) patients. A large number of evidence supporting that T2DM enhances BC risk [17] makes the notion of using metformin as a cancer prevention or treatment drug a very exciting prospect. Metformin activates the AMPK pathway by inhibiting complex 1 of the mitochondrial respiratory chain, resulting in the suppression of mTOR and hence loss of cell proliferation and repression of glucose synthesis [18]. It is noteworthy that metformin has been reported to decrease the incidence of cancer [19, 20]. The survival rate of BC patients treated with metformin is significantly higher than that of patients without metformin treatment.

As detailed previously, metformin exhibits unique anti-TNBC actions both in vitro and in vivo [21]. Metformin (8–14 mg/day) significantly reduces tumor growth in the TNBC MDA-MB-231 xenograft mouse model compared with the untreated control. Moreover, pretreatment with metformin (8–14 mg/day) before injection of MDA-MB-231 cells dramatically reduces tumor growth and incidence. Indeed, studies have clearly demonstrated that in terms of apoptosis, TNBC cell lines are more sensitive to metformin when compared to non-TNBC cell lines [21]. In view of its anti-TNBC activity in vitro and in vivo, metformin should be explored as a drug for the treatment of this aggressive type of BC. Nevertheless, there are still many unresolved problems and contradictory opinions in metformin used for TNBC treatment. Although metformin has good potential in vitro for the treatment of TNBC, these reports have not been substantiated in clinical studies, even though there is a tendency to decreased distant metastasis [22]. These contradictory conclusions may be explained by the following reasons: (1) The optimal concentration of metformin in laboratory models is higher than the conventional anti-diabetic dose, which may lead to the failure of this exposure level in the clinic; (2) Preclinical models have shown more obvious AMPK phosphorylation/activation by metformin than the clinical studies.

### AICAR

AMPK pharmacologic activator 5-aminoimidazole-4-carboxamide ribose (AICAR) is an analog of AMP and extensively used to stimulate AMPK in experiments. The mechanism that AICAR activates AMPK is thus different from that of metformin. AICAR can regulate cellular energy metabolism and induces mitochondrial proliferation and apoptosis. It has been demonstrated that AICAR has anti-cancer effects in many cancers [23–25]. Nevertheless, the effects of AICAR on BC, especially TNBC, and the roles of AMPK have been rarely reported.

More recently, studies have highlighted the role of metadherin (MTDH) in stimulating tumor progression, metastasis, and drug resistance in various cancers. MTDH is an oncogene that was originally identified as a gene increased in astrocytes treated with TNFα or infected with HIV1 [26]. MTDH mediates the oncogenic characteristics of PI3K, Ha-Ras, and c-Myc and influences cell survival and proliferation by activating Akt [27]. MTDH has also been reported to induce tumor cell metastasis via activating NF-κB [28]. A recent study has shown that elevated MTDH levels are associated with drug resistance through the induction of multidrug resistance protein 1 (MDR1) expression in hepatocellular carcinoma (HCC) [29]. MTDH is also involved in tumor angiogenesis [30]. A study from Gollavilli et al. [31] revealed a new regulatory mechanism for MTDH expression and demonstrated that AICAR, by stimulating GSK3β and SIRT1, decreases MTDH expression via suppressing c-Myc in TNBC cells. This action of AICAR is related to AMPK activation. Accordingly, AICAR, via AMPK activation, promotes growth arrest and anti-proliferative effects and inhibits migration/invasion of TNBC cells. Taken together, these findings elucidate a novel role of AMPK in regulating oncoproteins. The use of AMPK activators might substantiate to be beneficial, at least as an adjuvant therapy combined with other chemoprevention strategies, where MTDH has been associated with TNBC progression.

### RL71

Autophagy is a conserved catabolism process that delivers cytoplasmic constituents and organelles for degradation in the lysosome [32]. It can be triggered by various stressful conditions, e.g., nutrient deficiency, oxidative stress, and endoplasmic reticulum stress. In fact, overactivation of autophagy may ultimately cause type II programmed cell death in tumors [33]. One of the most important mechanisms of many clinically approved drugs or experimental small molecules with potential anti-cancer activity is the ability to induce autophagic cell death [34]. Induction of autophagic cell death can provide another way to treat cancer besides inducing apoptosis. Of note, recent studies have shown that the expression of autophagy-associated markers LC3/Beclin-1 in TNBC is the highest among BCs, indicating a constitutive activation of autophagy in TNBC [35]. Considering the threshold effect of autophagy differentiating survival and death in tumor cells, it is evident that further promoting autophagy with small molecular inducers can be used as a new TNBC therapy strategy.

More recently, RL71, a second-generation curcumin analog, has been shown to possess effective anti-cancer activity on TNBC cells through triggering excessive autophagic cell death [36]. RL71 increases the release of Ca^2+^ from the endoplasmic reticulum into the cytosol through repressing sarco/endoplasmic reticulum calcium-ATPase 2 (SERCA2) activity. Calcium signaling can promote autophagy via multiple mechanisms [37]. Here, The calcium mobilization induced by RL71 treatment results in the Ca^2+^/calmodulin-dependent kinase kinase-β (CaMKKβ)-dependent activation of AMPK, which represses the activity of the mTOR, a negative regulator of autophagy. Pharmaceutical inhibition of either CaMKKβ or AMPK decreases the conversion of LC3B-I to LC3B-II and inverts RL71-induced cell death in MDA-MB-468 cells. Accordingly, in TNBC xenograft mouse models, RL71 significantly inhibits tumor growth, reduces metastasis, and prolongs survival time. Together, these results indicate that AMPK is a potential therapeutic target candidate for TNBC and support the notion that autophagy inducers can be used as new therapies in TNBC treatment.

### Demethoxycurcumin

Recent research has focused on targeting metabolic pathways that may change during the initiation and development of TNBC. The inhibition of cancer cell growth through activating AMPK has attracted much attention. A study from Shieh et al. [38] investigating the effects of curcuminoids on the viability of TNBC cells suggests that demethoxycurcumin (DMC) at low micromolar levels potently represses TNBC cell proliferation by simultaneously inhibiting various oncogenic signaling pathways and energy metabolism via AMPK activation.

DMC is a structural analog of curcumin, showing nearly the same biological action as curcumin, e.g., anti-oxidative, anti-inflammatory, anti-tumor, and anti-angiogenesis [39] activities. Compared with ER-(+) or HER2-overexpressing BC cells, DMC exhibits the most effective cytotoxic effects on TNBC cells. Conversely, normal human mammary cells are unaffected by DMC treatment. AMPK was recently demonstrated to integrate growth factor signaling with cellular metabolism via negatively regulating mTOR [18, 40]. The role of mTOR is associated with the regulation of mRNA translation through the eukaryotic initiation factor 4E-binding protein-1 (4E-BP1) in mammalian cells. In its hyper-phosphorylation form by mTOR, 4E-BP1 eventually initiates translation of specific mRNAs, such as those required for cell cycle progression and those implicated in cell cycle regulation [41]. Indeed, signaling through DMC-induced AMPK activation is able to block 4E-BP1 signaling and mRNA translation through mTOR and inhibit the activity/expression of lipogenic enzymes, e.g., fatty acid synthase (FASN) and acetyl-CoA carboxylase (ACC).

Potential approaches in TNBC treatment include targeting EGFR, which is demonstrated to be crucial for the growth and maintenance of TNBC [42]. Of note, nearly 60% of basal-like TNBC expresses EGFR, and higher intratumoral expression of EGFR represents a poor prognosis, indicating EGFR as a molecular target for innovative therapeutic inhibitors [42]. Hence, inhibition of EGFR plays an important role in the phenotypic changes of TNBC [43]. DMC-mediated AMPK activation promotes EGFR degradation through regulating expression of the phosphatases, protein phosphatase 2A(PP2A) and SHP-2, in TNBC cells.

In addition to the aforementioned signaling pathways, DMC also targets various AMPK downstream targets. Among these, the dephosphorylation of Akt is notable since it avoids the feedback activation of Akt induced by mTOR inhibition. Furthermore, DMC inhibits lipopolysaccharides (LPS)-induced IL-6 production, thus hindering subsequent STAT3 activation. Together, these findings indicate that DMC is an effective AMPK agonist that acts through a wide range of anti-TNBC activities, and provide proof-of-concept that targeting AMPK represents a novel strategy for TNBC prevention and treatment.

### Fluoxetine

Fluoxetine, a selective serotonin reuptake inhibitor (SSRI), is widely used to regulate serotonin concentration in the central nervous system [44]. It is also commonly known as Prozac, which is used to treat depression. Of interest, there is encouraging evidence to support the contribution of Fluoxetine to inhibition of mitochondrial function leading to autophagic flux and apoptosis [45]. As detailed recently [46], Fluoxetine has robust anti-proliferative activities and triggers autophagic cell death in TNBC cells. The mechanism underlying Fluoxetine-induced autophagic cell death is related to activation of AMPK-mTOR-ULK complex axis and suppression of eukaryotic elongation factor-2 kinase (eEF2K). eEF2K, well-known as a Ca^2+^ calmodulin (CaM)-dependent kinase, is overexpressed in many cancers, especially TNBC [47]. Mounting evidence demonstrates that eEF2K can regulate the expression of various apoptotic proteins including Bcl-XL, XIAP, c-FLIP_L_, PI3KCI, and p70S6K to impede apoptosis in the tumor. Alternatively, eEF2K regulates autophagy implicated in AMPK, mTORC1, and ULK1, thus promoting tumor cell survival [48]. In addition, eEF2K may play an important role in the crosstalk between autophagic and apoptotic processes in TNBC.

In summary, these findings suggest that Fluoxetine effectively inhibits tumor growth of TNBC via promoting apoptosis and autophagy associated with suppression of eEF2K and activation of the AMPK-mTOR-ULK signaling pathway. These results have also led to the conclusion that AMPK may be a novel anti-TNBC target, and activating AMPK/inhibiting eEF2K will be a promising therapeutic strategy for TNBC.

### miR-200a

MicroRNA (miRNA) replacement therapy represents a promising way to target cancer pathways. miRNAs are small non-coding RNAs that have the ability to act as cancer suppressors and are commonly lost in several cancers [49]**.** Since miRNAs typically target various genes and pathways concurrently, an important benefit of miRNA replacement therapy is a lower resistance. The miR-200 family (miR-200a, -200b, -200c, -141, -429) is emerging as important cancer suppressor miRNAs [50]. Decreased expression of the miR-200 family is observed in TNBC and correlated with epithelial-to-mesenchymal transition (EMT), cancer progression, and an aggressive cancer phenotype [51]. miR-200a impedes EMT through targeting the E-cadherin suppressors *ZEB1/2* or *SUZ12,* leading to augmented levels of E-cadherin [52]**.** Since the reduction of E-cadherin expression is a feature of the TNBC subgroup [53] and these miRNAs are reduced in TNBC cells, miR-200 replacement therapy is an interesting option for future TNBC therapy.

More recently, miR-200a has been shown to reduce cell migration of TNBC, through its downregulation of the oncogene EPH receptor A2 (*EPHA2*) and subsequent activation of AMPK [54]. EPHA2 binds ephrin-A ligands on the cell membrane and modulates cell–cell interaction. Ephrin-A1-EPHA2 binding leads to receptor degradation and inhibition of migration and proliferation, while EPHA2 accumulates and induces invasiveness in the absence of ligand [55]. Multiple clinical data indicate a tumorigenic role for EPHA2 in BC development [56]. Of note, EPHA2 is overexpressed in TNBC cells whereas the ligand Ephrin-A1 is undetectable; hence, EPHA2 plays an important role in promoting TNBC invasion [57]. Thus, EPHA2 has been considered as a promising therapeutic target for TNBC. Actually, the expression of *EPHA2* is associated with the poor survival rate of basal-like BC and that its expression is inhibited by miR-200a by directly interacting with the 3′UTR of *EPHA2* [54]*.* Therefore, it is evident that the miR-200a-EPHA2-AMPK axis is a new mechanism highlighting the significance of utilizing AMPK activation to target TNBC metastases.

### OSU-53

Lee et al. developed a lead AMPK-activating compound OSU-53 by using ciglitazone as a scaffold on the basis of the finding that thiazolidinediones induce AMPK activation partially through a peroxisome proliferator-activated receptor (PPAR)γ-independent mechanism [58]**.** OSU-53 is a PPARγ-inactive derivative, which stimulates the activity of AMPK kinase by efficient direct activation. This mechanism is different from that of metformin or AICAR.

OSU-53 potently inhibits TNBC by simultaneously hindering various oncogenic signaling pathways and energy metabolism as a result of AMPK activation [58]. Among those, the PP2A-dependent dephosphorylation of Akt is important since it avoids the feedback activation of Akt caused by mTOR inhibition. OSU-53 also regulates energy homeostasis by inhibiting fatty acid (FA) biosynthesis and shifting the metabolism to oxidation through increasing the expression of primary regulatory factors of mitochondrial biogenesis, including PPARγ coactivator 1α (PGC-1α) and the transcription factor nuclear respiratory factor 1. Additionally, OSU-53 inhibits LPS-induced IL-6 production, thus hindering subsequent activation of STAT3. It is also noteworthy that OSU-53 suppresses hypoxia-induced EMT through decreasing the expression of hypoxia-inducible factor 1-alpha (HIF1α) and the E-cadherin repressor Snail. In spite of its extensive anti-cancer activities, OSU-53 does not significantly affect non-malignant MCF-10A cells, partially owing to the low basal activation levels of Akt/mTOR. Importantly, oral OSU-53 inhibits TNBC xenograft tumor growth without overt toxicity. This in vivo efficacy, together with the wide range of anti-cancer properties of OSU-53, provides proof-of-concept that AMPK is an important therapeutic target for TNBC.

In addition to the AMPK activators mentioned above, polyphenols have also been shown to inhibit TNBC by activating AMPK. A combined diet of grape polyphenols comprising of resveratrol, quercetin, and catechin (RQC) impedes TNBC growth and metastasis in nude mice. Furthermore, RQC sensitizes TNBC tumors to the anti-EGFR therapeutic gefitinib, through activation of AMPK and subsequent suppression of the Akt/mTOR pathway [59]. Of note, this study further substantiated the relative contribution of AMPK activation to the effects of RQC on mTOR pathway, concomitant with a decrease in the TNBC progression, which is further testament to the significance of targeting AMPK in the TNBC therapy.

### AMPK inhibition in TNBC treatment

AMPK activation in response to stress is associated with augmented resistance to death in many cellular systems. Thus, the effects of AMPK activation in cancer seem much more complicated than originally thought, and AMPK can act as a cancer “friend” or “enemy” in specific circumstances. AMPK supra-physiological activation induced by drugs decreases cancer growth in vitro and in preclinical models by inhibiting crucial biosynthetic pathways [60, 61]. Nevertheless, AMPK’s physiological activation upon various stresses, such as hypoxia and glucose deprivation, provides metabolic adaptation for tumor cells to survive metabolic stress [62]. Upon glucose depletion, AMPK is activated and harmoniously supports cell survival through multiple mechanisms including (1) induction of autophagy [63], (2) promotion of fatty acid oxidation to produce ATP [64, 65], (3) transcriptional alterations induced by the core histone H2B phosphorylation [66], and (4) elevation of intracellular NADPH levels to combat cytotoxic ROS [67]. Therefore, although the LKB1/AMPK pathway can be considered as a tumor suppressor, it can also act as a “tumor-promoting factor,” making cancer cells more resistant to metabolic stress. In view of the complex roles of AMPK under different metabolic conditions, the role of AMPK in cancer should be reconsidered carefully.

In our own laboratory, we demonstrated that DNA damage (UV or bleomycin), via PARP-1 activation, induces ATP depletion at earlier times, which results in AMPK activation and ATP increase at later times of DNA damage. Treating cancer cells with the AMPK inhibitor, compound C, or overexpression of a dominant-negative mutant of AMPK (DN-AMPK) blocked elevation of ATP levels at later times of DNA damage, causing significant cell apoptosis [68]. More recently, our findings [69] indicated that 2-deoxyglucose (2-DG), a glycolytic inhibitor, activates AMPK, followed by a partial restore of intracellular ATP levels. Thus, the inhibitory effect of 2-DG on cancer cell growth is partially offset by the fact that there is also 2-DG-induced AMPK activation. Of note, compound C, an AMPK inhibitor, synergistically reinforced the inhibitory effect of 2-DG on ATP production and caused a dramatic increase in cytotoxicity in breast cancer cells but not in normal MCF-10A cells. The killing effect of this 2-DG/compound C combination treatment on TNBC cells is more obvious than on MCF-7. Together, we demonstrate that combination treatment of DNA damaging therapeutic agents or 2-DG and AMPK inhibitors is a potentially promising, safe, and effective breast cancer treatment strategy.

In short, these evidently contradictory results indicate that both AMPK agonists and antagonists may provide therapeutic benefits in distinct cancer types, genetic/metabolic circumstances, and micro-environmental conditions. Therefore, the choice of AMPK regulators might be different at different stages of carcinogenesis or cancer progression, or in different treatment contexts.

### Translational challenges

TNBCs are predominantly sensitive to blockade of the mTOR pathway. The mTOR signal passes through two multi-protein complexes, i.e., mTORC1 and mTORC2 (Fig. 1). A study [14] using RNA interference experiments to determine the contribution of each of the two complexes to the modulation of TNBC cell number suggests that mTORC1 is superior to mTORC2 in controlling TNBC cell proliferation. Additionally, the RNA interference of mTOR has a better anti-proliferative effect than that of mTOR1 or mTOR2 alone. Thus, mTOR targeting could be a more effective anti-TNBC treatment versus solely acting on the mTORC1 pathway. This is relevant as the majority of mTOR inhibitors used in the clinic functions on mTORC1.

Although targeting AMPK/mTOR has become a potential target for cancer treatment, in some cases, AMPK activation might promote cancer. A majority of tumors have activation in mTORC1, which promotes growth through effectors including 4E-BP1 and S6K1, as mentioned above. Therefore, inhibition of mTORC1 will thwart cell protein synthesis and proliferation; nonetheless, as mentioned earlier, the inhibition of mTORC1 without impeding mTORC2 may result in compensatory stimulation of PI3K/Akt signaling pathway and promotion of cancer survival. In most cases, AMPK activation leads to mTORC1 repression; but the effects on mTORC2/Akt activation are not entirely clear. If a specific AMPK activator is only used to modulate mTOR, its success will depend on its ability to inhibit the two mTOR branches. AMPK agonists have the ability to inhibit or promote tumors, which may be due to feedback-related mechanisms under specific circumstances. Therefore, the biggest translational challenge of exploiting AMPK as a therapeutic target and developing AMPK agonists is how to promote the anti-cancer effect and circumvent pro-cancer effects.

## Conclusions

**Fig. 2:**
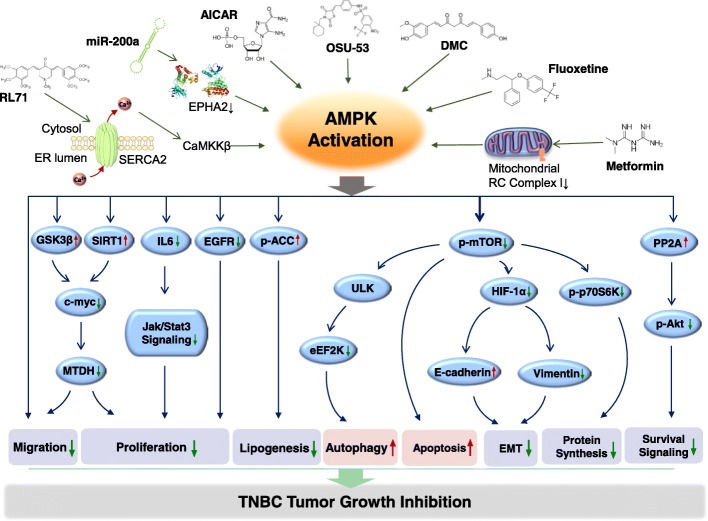
Proposed molecular action of AMPK activation by various classical/novel AMPK activators in TNBC. Abbreviations: DMC, demethoxycurcumin; AICAR, 5-aminoimidazole-4-carboxamide ribose; SERCA2, sarco/endoplasmic reticulum calcium-ATPase 2; CaMKKβ, Ca2+/calmodulin-dependent kinase kinase-β; RC: respiratory chain; EPHA2, EPH receptor A2; MTDH, metadherin

Substantial evidence supports the idea that AMPK activation may be used as a metabolic cancer suppressor. We can presume that AMPK activation may counteract the development of cancer through reprogramming cellular metabolism and targeting one of the essential components necessary for tumor progression. In fact, AMPK function is compromised in primary BCs and loss of AMPK signaling is related to a worse clinical outcome, implicating that AMPK reactivation has the potential for prevention and treatment in BC. Recently, the possible role of an abnormal AMPK signaling pathway in the growth, survival, and drug resistance of TNBC has been well understood. Of pertinence to this review, various AMPK agonists such as metformin, AICAR, RL71, DMC, Fluoxetine, miR-200a, and OSU-53 were demonstrated to significantly inhibit TNBC. Activation of AMPK has positive effects in TNBCs because of its targeting inhibition effect on Akt/mTOR. Additionally, AMPK activation suppresses EGFR, c-Myc, IL6, Jak/STAT3, HIF-1α, and eEF2K, which are well-established hallmarks of TNBCs. On the other hand, under certain circumstances, such as in combination with DNA damaging therapeutic agents or glycolytic inhibitors, inhibition of AMPK can also be used as a means to treat TNBC. Therefore, it is plausible to consider AMPK as an attractive therapeutic target for TNBC (Fig. 2).

Future preclinical and clinical studies would have to further substantiate the anti-TNBC effects of AMPK activation by using more specific, direct, and potent AMPK activators. In addition, all other indirect effects of AMPK activation remain to be studied. Hence, additional studies are needed before AMPK activators can be used for clinical treatment of TNBC. At the time of writing this review, more than 100 patents have been filed for new small molecule AMPK agonists, and it is hoped that some of them will soon enter clinical trials. It seems possible that within 5–10 years, we will know more clearly whether a more selective and potent AMPK activator than metformin will have a place in the treatment of TNBCs.

## Abbreviations

4E-BP1: eIF4E-Binding protein-1
ACC: Acetyl-CoA carboxylase
AICAR: 5-Aminoimidazole-4-carboxamide ribose
AMPK: AMP-activated protein kinase
AR: Androgen receptor
BC: Breast cancer
CaMKKβ: Ca2+/calmodulin-dependent kinase kinase-β
DFS: Disease-free survival
DMC: Demethoxycurcumin
eEF2K: Eukaryotic elongation factor-2 kinase
EGFR: Epidermal growth factor receptor
EMT: Epithelial-to-mesenchymal transition
*EPHA2*: EPH receptor A2
ER: Estrogen receptor
FA: Fatty acid
FASN: Fatty acid synthase
HCC: Hepatocellular carcinoma
HER2: Human epidermal growth factor receptor-2
HIF1α: Hypoxia-inducible factor 1-alpha
LKB1: Liver kinase B1
MAPK: Mitogen-activated protein kinase
miRNA: MicroRNA
MTDH: Metadherin
mTOR: Mammalian target of rapamycin
NTNBC: Non-triple-negative breast cancer
OS: Overall survival
PARP: Poly [ADP-ribose] polymerase
PGC-1α: PPARγ coactivator 1α
PKC: Protein kinase C
PP2A: Protein phosphatase 2A
PPAR: Peroxisome proliferator-activated receptor
PR: Progesterone receptor
RAPTOR: Regulatory-associated protein of mammalian target of rapamycin
RICTOR: Rapamycin-insensitive companion of mTOR
RQC: Resveratrol, quercetin, and catechin
SERCA2: Sarco/endoplasmic reticulum calcium-ATPase 2
SGK: Serum/glucocorticoid-regulated kinase
SSRI: Selective serotonin reuptake inhibitor
STAT3: Signal transducer and activator of transcription 3
T2DM: Type 2 diabetes mellitus
TNBC: Triple-negative breast cancer
VEGFR: Vascular endothelial growth factor receptor
